# Supplementary material for: Unveiling the Bioactive Potential of the Invasive Jellyfish Phyllorhiza punctata Through Integrative Transcriptomic and Proteomic Analyses
Source: Biomolecules. 2025 Aug 4;15(8):1121. doi: 10.3390/biom15081121 (PMC12383608; doi:10.3390/biom15081121)
Supplement: Supplementary file 1 [file biomolecules-15-01121-s001.zip › Suplementary Material/FileS3/PEPFOLD4/XP_065068415_14880/clusters.html]

 PEP-FOLD clusters


Download archive of all models  here   

|  |  |  |
| --- | --- | --- |
| # | file | sOPEP |
| # cluster 1 - Size: 15 |  |  |
|  | XP\_065068415\_14880-model1 | -4.92467 |
|  | XP\_065068415\_14880-model1.1 | -4.92467 |
|  | XP\_065068415\_14880-model1.2 | -4.92467 |
|  | XP\_065068415\_14880-model1.3 | -4.92467 |
|  | XP\_065068415\_14880-model1.4 | -4.92059 |
|  | XP\_065068415\_14880-model1.5 | -4.91757 |
|  | XP\_065068415\_14880-model1.6 | -4.91341 |
|  | XP\_065068415\_14880-model1.7 | -4.86853 |
|  | XP\_065068415\_14880-model1.8 | -4.86575 |
|  | XP\_065068415\_14880-model1.9 | -4.85167 |
|  | XP\_065068415\_14880-model1.10 | -4.84254 |
|  | XP\_065068415\_14880-model1.11 | -4.81926 |
|  | XP\_065068415\_14880-model1.12 | -4.78113 |
|  | XP\_065068415\_14880-model1.13 | -4.71423 |
|  | XP\_065068415\_14880-model1.14 | -4.57311 |
| # cluster 2 - Size: 6 |  |  |
|  | XP\_065068415\_14880-model2 | -3.51139 |
|  | XP\_065068415\_14880-model2.1 | -3.47051 |
|  | XP\_065068415\_14880-model2.2 | -3.46151 |
|  | XP\_065068415\_14880-model2.3 | -3.45791 |
|  | XP\_065068415\_14880-model2.4 | -3.44488 |
|  | XP\_065068415\_14880-model2.5 | -3.43416 |
| # cluster 3 - Size: 40 |  |  |
|  | XP\_065068415\_14880-model3 | -3.48377 |
|  | XP\_065068415\_14880-model3.1 | -3.47926 |
|  | XP\_065068415\_14880-model3.2 | -3.45605 |
|  | XP\_065068415\_14880-model3.3 | -3.45553 |
|  | XP\_065068415\_14880-model3.4 | -3.45553 |
|  | XP\_065068415\_14880-model3.5 | -3.43984 |
|  | XP\_065068415\_14880-model3.6 | -3.43984 |
|  | XP\_065068415\_14880-model3.7 | -3.43984 |
|  | XP\_065068415\_14880-model3.8 | -3.43984 |
|  | XP\_065068415\_14880-model3.9 | -3.43984 |
|  | XP\_065068415\_14880-model3.10 | -3.43984 |
|  | XP\_065068415\_14880-model3.11 | -3.43984 |
|  | XP\_065068415\_14880-model3.12 | -3.43984 |
|  | XP\_065068415\_14880-model3.13 | -3.43511 |
|  | XP\_065068415\_14880-model3.14 | -3.43511 |
|  | XP\_065068415\_14880-model3.15 | -3.43511 |
|  | XP\_065068415\_14880-model3.16 | -3.43511 |
|  | XP\_065068415\_14880-model3.17 | -3.43407 |
|  | XP\_065068415\_14880-model3.18 | -3.43153 |
|  | XP\_065068415\_14880-model3.19 | -3.43102 |
|  | XP\_065068415\_14880-model3.20 | -3.41209 |
|  | XP\_065068415\_14880-model3.21 | -3.41012 |
|  | XP\_065068415\_14880-model3.22 | -3.40743 |
|  | XP\_065068415\_14880-model3.23 | -3.40311 |
|  | XP\_065068415\_14880-model3.24 | -3.40311 |
|  | XP\_065068415\_14880-model3.25 | -3.40311 |
|  | XP\_065068415\_14880-model3.26 | -3.40311 |
|  | XP\_065068415\_14880-model3.27 | -3.40244 |
|  | XP\_065068415\_14880-model3.28 | -3.39635 |
|  | XP\_065068415\_14880-model3.29 | -3.39635 |
|  | XP\_065068415\_14880-model3.30 | -3.39144 |
|  | XP\_065068415\_14880-model3.31 | -3.38623 |
|  | XP\_065068415\_14880-model3.32 | -3.38205 |
|  | XP\_065068415\_14880-model3.33 | -3.38205 |
|  | XP\_065068415\_14880-model3.34 | -3.38205 |
|  | XP\_065068415\_14880-model3.35 | -3.37739 |
|  | XP\_065068415\_14880-model3.36 | -3.37643 |
|  | XP\_065068415\_14880-model3.37 | -3.37159 |
|  | XP\_065068415\_14880-model3.38 | -3.36708 |
|  | XP\_065068415\_14880-model3.39 | -3.36124 |
| # cluster 4 - Size: 24 |  |  |
|  | XP\_065068415\_14880-model4 | -3.4814 |
|  | XP\_065068415\_14880-model4.1 | -3.46596 |
|  | XP\_065068415\_14880-model4.2 | -3.46589 |
|  | XP\_065068415\_14880-model4.3 | -3.46514 |
|  | XP\_065068415\_14880-model4.4 | -3.44728 |
|  | XP\_065068415\_14880-model4.5 | -3.43254 |
|  | XP\_065068415\_14880-model4.6 | -3.42707 |
|  | XP\_065068415\_14880-model4.7 | -3.42383 |
|  | XP\_065068415\_14880-model4.8 | -3.42383 |
|  | XP\_065068415\_14880-model4.9 | -3.42182 |
|  | XP\_065068415\_14880-model4.10 | -3.42084 |
|  | XP\_065068415\_14880-model4.11 | -3.42084 |
|  | XP\_065068415\_14880-model4.12 | -3.41842 |
|  | XP\_065068415\_14880-model4.13 | -3.41807 |
|  | XP\_065068415\_14880-model4.14 | -3.41486 |
|  | XP\_065068415\_14880-model4.15 | -3.41369 |
|  | XP\_065068415\_14880-model4.16 | -3.40456 |
|  | XP\_065068415\_14880-model4.17 | -3.40439 |
|  | XP\_065068415\_14880-model4.18 | -3.40288 |
|  | XP\_065068415\_14880-model4.19 | -3.39743 |
|  | XP\_065068415\_14880-model4.20 | -3.37756 |
|  | XP\_065068415\_14880-model4.21 | -3.36893 |
|  | XP\_065068415\_14880-model4.22 | -3.35652 |
|  | XP\_065068415\_14880-model4.23 | -3.34202 |
| # cluster 5 - Size: 13 |  |  |
|  | XP\_065068415\_14880-model5 | -3.47741 |
|  | XP\_065068415\_14880-model5.1 | -3.43794 |
|  | XP\_065068415\_14880-model5.2 | -3.43311 |
|  | XP\_065068415\_14880-model5.3 | -3.43311 |
|  | XP\_065068415\_14880-model5.4 | -3.43311 |
|  | XP\_065068415\_14880-model5.5 | -3.43311 |
|  | XP\_065068415\_14880-model5.6 | -3.43311 |
|  | XP\_065068415\_14880-model5.7 | -3.43311 |
|  | XP\_065068415\_14880-model5.8 | -3.42342 |
|  | XP\_065068415\_14880-model5.9 | -3.40507 |
|  | XP\_065068415\_14880-model5.10 | -3.40507 |
|  | XP\_065068415\_14880-model5.11 | -3.38471 |
|  | XP\_065068415\_14880-model5.12 | -3.35565 |
| # cluster 6 - Size: 2 |  |  |
|  | XP\_065068415\_14880-model6 | -3.44412 |
|  | XP\_065068415\_14880-model6.1 | -3.39528 |
|  |  |  |
